# Simple intrapleural hyperthermia at thoracoscopic exploration to treat malignant pleural effusion

**DOI:** 10.1186/s13019-015-0340-8

**Published:** 2015-10-28

**Authors:** Youngkyu Moon, Kyung Soo Kim, Jae Kil Park

**Affiliations:** Department of Thoracic & Cardiovascular Surgery, Seoul St. Mary’s Hospital, Catholic Medical Center School of Medicine, The Catholic University of Korea, 222 Banpo-daero, Seoul, Seocho-gu 06591 Republic of Korea

**Keywords:** Pleural effusion, Malignant, Thoracoscopy, Pleurodesis

## Abstract

**Background:**

Malignant pleural effusion (MPE) occurs at a terminal stage of cancer, and related symptoms may considerably reduce a patient’s respiratory function and quality of life. We assessed the benefit of simple intrapleural hyperthermia (SIH) during thoracoscopic exploration for MPE.

**Methods:**

We conducted a retrospective review of 34 patients underwent thoracoscopic exploration and SIH procedures for MPE between April, 2009 and July, 2014 at our institution. One month after removal of the tube, therapeutic efficacy was evaluated, calculating response rates and recurrence rate.

**Results:**

In this cohort (male, 11; female, 23; average age, 54.2 ± 12.7 years), the most frequent primary cancers were breast (*n* = 11, 32.4 %), lung (*n* = 10, 29.4 %), and ovarian (*n* = 6, 17.6 %). Therapeutic response (ie, presence of pleural effusion) was assessed 1 month after chest tube removal, with 19 (55.9 %) showing complete response (CR), 9 (26.5 %) showing partial response (PR), and non-response (NR) seen in 6 (17.6 %). The combined (CR + PR) response rate was 82.4 %. During follow-up, there were seven instances of recurrence, requiring repeat drainage. Three- and 7-month recurrence-free rates were 86.9 and 73.9 %, respectively. No postoperative respiratory complications or fever developed. Early death within 3 months from progression of primary cancer was identified as a risk factor in patients of NR status (HR = 18.36, *p* = 0.043).

**Conclusions:**

If thoracoscopic exploration is indicated for MPE, SIH is a safe and effective management alternative in patients whose primary malignancy is not rapidly progressing.

## Background

Malignant pleural effusion (MPE) often occurs in the course of malignancies and may impair pulmonary function. Quality of life is also diminished due to symptoms (ie, dyspnea, pain, cough, etc.) [1]. Various cancers (lung, breast, or ovarian) and hematogenous neoplasms (lymphomas) often culminate in MPE, although in many instances, the source is unknown [1, 2]. However, lung and breast cancers are implicated in > 50 % of such cases, according to reports [3].

MPE develops when malignant cells metastasize to pleura, but diagnostic cells may or may not be detectable in pleural fluid. On the other hand, paramalignant pleural effusion does occur in the course of some cancers and is not easily differentiated from MPE [1, 2]. For any pleural effusion found in a patient with confirmed malignancy, fluid cytology is normally requested. If cytology is unproductive, pleural biopsy may be needed, usually via diagnostic thoracoscopic exploration. Pleural biopsy is sometimes the only recourse in instances where primary tumors are unknown [4, 5].

Thoracoscopic exploration enables direct identification of pleural nodules, with biopsy of pleura and lung for pathologic confirmation. Even if MPE is documented, thoracoscopic exploration also serves a therapeutic purpose, allowing more effective drainage of any loculated fluid. When done for above reasons, pleurodesis is typically performed as well, instilling sclerosing agents at the close of exploration. The latter is especially warranted in patients faced with long-term tube placement for recurrent pleural effusion.

Although talc is generally the most effective agent for pleurodesis, serious complications, such as acute respiratory distress syndrome (ARDS) and systemic inflammation, may result. Graded talc is used in the United States and in Europe to minimize complications [6] However, availability is limited in Asia. Other agents, such as doxycycline, bleomycin, iodopovidone, and silver nitrite, are less effective than talc, and related studies are few [7].

Some reports indicate that tumor cell survival may be reduced by exposure to solutions at supraphysiologic temperatures [8–11]. Intrapleural hyperthermic chemotherapy has been used to treat pleural malignancy or pleural metastasis of lung cancer. By combining a chemotherapeutic agent with a hyperthermic solution, survival times may actually be increased [12–15]. However, almost no accounts to date describe the use of simple intrapleural hyperthermia (SIH) for MPE.

Consequently, we attempted to confirm the benefits of SIH in patients where MPE was diagnosed or strongly suspected, necessitating thoracoscopic exploration for diagnosis or for drainage of fluid. Our aim was to achieve immediate symptom improvement and maintain stability long-term, avoiding recurrences. Other objectives were minimization of complications, shortening of hospitalization periods, and cost-effectiveness [1].

## Methods

### Patients

Among 45 patients who underwent SIH procedures after thoracoscopic exploration in the period between April, 2009 and July, 2014, three patients were lost to follow-up, four patients had pleural metastasis without pleural effusion, and four patients had benign pleural biopsies with no primary cancers. The latter were excluded, leaving 34 patients who qualified for retrospective chart review. In all patients, pleural effusion was confirmed by computed tomography (CT) of the chest. Thoracoscopic pleural biopsy was done in patients with medical histories of primary cancers and suspected pleural metastases or with no primary cancer by history but with suspected MPE. In instances where MPE was already confirmed by fluid cytology, thoracoscopic drainage of loculated pleural fluid was opted. SIH was conducted following thoracoscopic procedures. All of the patients consented the procedure before operation. The study was approved by the institutional Review Board of Seoul St. Mary’s Hospital (The Catholic University of Korea)

### Surgical technique

**Fig. 1:**
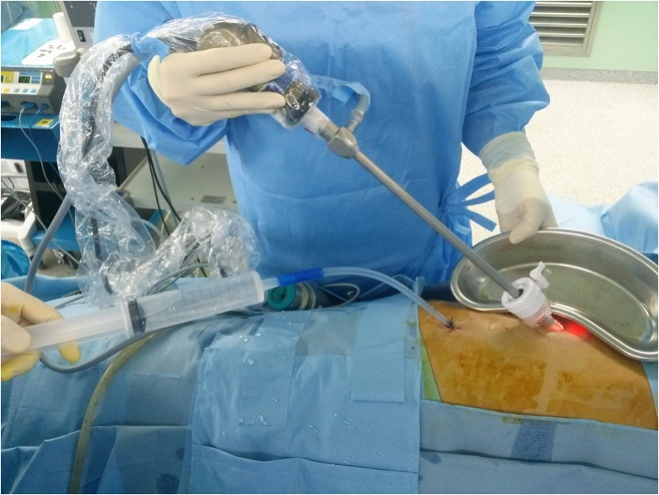
Normal saline (48 °C) injection through the chest tube under thoracoscopic observation

**Fig. 2:**
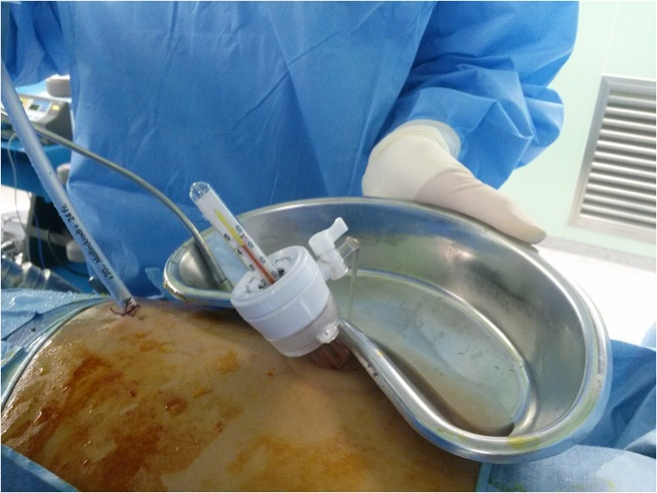
Leaving the port open for natural drainage of saline and continuously monitoring the temperature of effluent fluid using bar thermometer

Under general anesthesia and with patients in lateral decubitus position, two ports (10 mm and 5 mm) were created at points where pleural effusions were most voluminous. After completing thoracoscopic pleural biopsy or pleurolysis (for loculated effusion), a chest tube was inserted through one port, reaching the deepest point in thoracic cavity, and SIH was conducted. With the thoracoscope deployed through the other port, normal saline (48 °C) was continuously injected through the chest tube under observation (Fig. 1). We used 48 °C saline because of rapid decline of saline temperature due to low velocity of manual infusion flow. Once the thoracic cavity was completely filled with saline, the thoracoscope was removed, leaving the port open for natural drainage of saline. During this time, a bar thermometer inserted through thoracoscopic port served to continuously monitor temperature inside the thoracic cavity (Fig. 2). Our SIH method involved frequent injection of physiologic saline solution (48 °C, 30 min) into thoracic cavity via chest tube, keeping pleural temperature above 42 °C. After 30 min, all intrathoracic saline solution was removed by suction, and the wound was closed with a chest tube in place.

### Chest tube removal

Tubes remained in position for 2 days at minimum and were removed once the amount of drainage fluid was < 150 ml in a 24-h period.

### Treatment efficacy

Following all operative procedures, plain x-ray films of the chest were obtained daily. Chest x-ray and CT imaging were also done periodically after hospital discharge to monitor pleural effusion status. Criteria (WHO) for evaluating therapeutic effects in patients with solid tumors were modified to assess outcomes of treatment in this setting; Complete response (CR), partial response (PR), and non-response (NR) corresponded with absence of effusion for > 4 weeks after treatment, 50 % reduction in effusion for > 4 weeks after treatment, and no reduction in pleural effusion, respectively [16]. Any drainage required in follow-up was defined as recurrence [17].

### Statistical analysis

Data were expressed as mean values ± standard deviation (SD) or percentages. Operative mortality was defined as in-hospital mortality or death within 30 days after procedures. Response rate was calculated as the ratio between combined responses (CR + PR) and total patient count. Recurrence-free rate was calculated for specified time intervals, applying Kaplan-Meier method. Logistic regression analysis was used to determine factors linked with patient non-response (NR). Statistical significance was set at *p* < 0.05.

## Results

A total of 34 patients (mean age, 54.2 ± 2.7 years; male-to-female ratio, 11:23) with MPE submitted to thoracoscopic exploration at our institution. Breast (*n* = 11, 32.4 %), lung (*n* = 10, 29.4 %), and ovary (*n* = 6, 17.6 %) were the most common primary tumor sites. The remainder (*n* = 7, 20.6 %) included two malignancies of unknown origin, two hepatic cancers, and one instance each of primary renal, peritoneal, and pleura (mesothelioma) neoplasms. Procedures performed were unilateral (right: *n* = 16, 47.1 %; left: *n* = 10, 29.4 %) or bilateral (*n* = 8, 23.5 %). In 24 patients (70.6 %), malignancy was diagnosed via pleural biopsy or pleural fluid cytology. Malignancy was unproven in the other 10 patients (29.4 %), despite primary tumors in advanced stages and persistent pleural effusion. Most patients (*n* = 30, 88.2 %) required pleurocentesis prior to surgery (Table 1).

**Table 1:** Patients characteristics (*n* = 34)

Characteristic	Value
Age, years (mean ± SD)	54.2 ± 12.7
Gender, *n* (%)	
Male	11 (32.4 %)
Female	23 (67.6 %)
Primary malignancy, *n* (%)	
Lung	10 (29.4 %)
Breast	11 (32.4 %)
Ovarian	6 (17.6 %)
Other	7 (20.6 %)
Laterality, *n* (%)	
Right	16 (47.1 %)
Left	10 (29.4 %)
Bilateral	8 (23.5 %)
Histologic confirmation, *n* (%)	
Confirmed MPE	24 (70.6 %)
Unconfirmed MPE	10 (29.4 %)
Preoperative pleural drainage, *n* (%)	30 (88.2 %)

*SD* = standard deviation

*MPE* = Malignant pleural effusion

Thoracoscopic exploration was regularly performed in advance of SIH, which was sustained at 42°-45 °C for 30 min. Temperature of thoracic esophagus was measured during all operations, with maximum levels falling below 40 °C. Mean body temperature 30 min after surgery was 36.6 ± 0.3 °C, and no patients were febrile on the first postoperative day. Blood pressures (BP) taken mid-procedure were stable. Although mean heart rate (HR) was 99.5 ± 22.0 beats/min, HR < 100 beats/min were recorded within 30 min after all procedures. Chest tubes were kept in place for a minimum of 2 days postoperatively but were removed once drainage was < 150 ml/24 h. Mean duration of chest tube drainage was 9.2 ± 4.8 days. Patients were discharged from the hospital on the days following chest tube removal, if additional treatment for primary malignancy was not required.

No postoperative respiratory complications or fever were recorded. One patient with ovarian cancer and recurrent MPE died postoperatively on Day 35. In this instance, the chest tube was removed on Day 14 after SIH, but carcinomatosis peritonei was unrelenting and aspiration pneumonia developed on Day 27, leading to death.

Pleural effusions were confirmed by x-ray or CT of the chest one month after chest tube removal. There were 19 patients (55.9 %) of CR status, 9 patients (26.5 %) of PR status, and 6 patients (17.6 %) of NR status. The combined (CR + PR) response rate was 82.4 %. Mean follow-up period overall was 382.6 days (range, 35–1885 days). Seven patients (CR, 1; PR, 1; NR, 5) underwent repeat drainage of pleural effusions (recurrent MPE) during follow-up. Kaplan-Meier method was applied to calculate recurrence-free rate (86.9 % at 3 months; 73.9 % at 7 months) (Table 2). Logistic regression analysis was used to evaluate risk factor of NR. In multivariate analysis, along with age, malignant fluid cytology, and type of primary cancer, early death within 3 months showed a significant association with NR status (HR = 18.36; *p* = 0.043) (Table 3).

**Table 2:** Surgical outcomes

	*n* (%)
Postoperative complications	
Respiratory events	0 (0 %)
Fever	0 (0 %)
Operative mortality	1 (3 %)
Response rates	
Complete (CR)	19 (55.9 %)
Partial (PR)	9 (26.5 %)
None (NR)	6 (17.6 %)
Combined (CR + PR)	28 (82.4 %)
Recurrence rate	7 (20.6 %)
3-month recurrence-free survival	86.9 %
4-month recurrence-free survival	82.9 %
7-month recurrence-free survival	73.7 %

*CR* = complete response

*PR* = partial response

*NR* = no response

**Table 3:** Risk factors for non-response to treatment (multivariate analysis)

Factors	Hazard ratio	95 % confidence interval	*P* value
Age	1.030	0.940–1.129	0.522
Primary cancer	1.065	0.334–3.403	0.915
Malignant cells in pleural fluid	5.830	0.370–91.839	0.210
Death within 3 months	18.360	1.098–306.912	0.043

## Discussion

Patients with MPE often require management, given their declining general condition and quality of life. Most of their symptoms may be temporarily alleviated by pleurocentesis, and in instances where repeated drainage is required, other methods (eg, pleurodesis, indwelling catheter, and pleuroperitoneal shunt) are useful [3]. Although pleurodesis has been the preferred treatment, incorporating talc as a sclerosing agent to obliterate the pleural space [1, 2], an indwelling tunneled pleural catheter (TPC) is now used in many countries. Unlike pleurodesis, chemotherapy is not delayed by TPC use, so hospital stays are reduced; and results have been durable, similar to those achieved with talc [5, 18–20]. However, the inconvenience of managing a catheter at home, concerns of catheter-related infection, and less complete obstruction of re-accumulating fluid are drawbacks. As a rule, Western patients are more receptive to in-home management, whereas Korean patients are more inclined to be fully treated as inpatients and make less use of TPCs.

As the most effective agent available for pleurodesis [1, 3, 21, 22], talc may be administered through thoracoscopic poudrage or as a slurry injected via chest tube. A higher success rate is attached to a thoracoscopic approach [1]. Small-sized talc particles tend to disseminate systemically, often provoking an inflammatory response. Hence, the use of graded talc (with small particles removed) is advised [1, 2, 6, 23], although this product is not readily available in many countries, including Korea.

A number of talc alternatives, such as doxycycline, povidone iodine, and antineoplastic agents, have been tried without success at our facility. However, very few reports substantiate their efficacy, and related complications, such as pain, fever, and systemic inflammation, were no fewer when compared with talc. Thus, they are not considered acceptable for pleurodesis [1, 24].

Direct injection of chemotherapeutic agents into pleural metastases is also done at times for local control of malignancy. Reports have indicated that apoptosis of tumor cells is accelerated by exposure to supraphysiologic hyperthermic solution [8–11]. Until recently, the observed survival benefits were attributed to a combination of hyperthermic solution and chemotherapeutic agent injected into the thoracic cavity [12–15, 25]. However, such results were assured by performing local treatment under conditions of pleural metastasis exclusively (without systemic dissemination), and this approach has never been utilized to manage MPE. In our hospital, such treatments are for palliation only, so a trial of hyperthermic saline alone (without chemotherapy) was initiated for MPE management. Although study subjects had persistent pleural effusions, diagnoses were unconfirmed. Thoracoscopic surgery was therefore required to obtain pleural biopsies and/or drain loculated fluid. Ultimately, SIH (30 min) was conducted following thoracoscopic exploration.

MPE treatment outcomes have been evaluated in various ways by a number of researchers. Checking for recurrence of pleural effusion on follow-up chest x-ray or CT imaging is the usual means of assessing therapeutic response. The calculated recurrence rate is also based on the need to re-insert a chest tube or catheter to drain a growing pleural effusion [7]. For our purposes, we calculated CR, PR, and NR by evaluating treatment response 1 month after chest tube removal. Recurrence was assumed if drainage of pleural fluid was required during follow-up.

Our approach yielded satisfactory therapeutic effect, with an overall response rate of 82.4 % and recurrence-free rates of 90 % and 70 % at 3 and 7 months, respectively. Comparisons admittedly were imprecise, owing to the fact that assessment of response to treatment differed across studies. Assuming a response rate of 80-90 % for thoracoscopic talc poudrage and 60-80 % for talc slurry, the efficacy shown in this study was on par with other reports [7].

In the course of this study, no major complications, such as respiratory events and fever, were encountered, and there were no minor complications, except for pain early-on at chest tube insertion sites. Accordingly, SIH appears much safer than pleurodesis, showing far fewer problems than pleurodesis in terms of systemic inflammation, respiratory complications, and intense pain. Nevertheless, intensive monitoring is critical to maintain intrathoracic operative temperatures > 42 °C, while closely monitoring BP, HR, and EKG and keeping the patient’s body temperature <40 °C.

Other than the cost of thoracoscopic exploration, no added expense accrued for SIH. A solution of saline (at 48 °C) for simple, manual injection into thoracic cavity via chest tube was the sole requirement. However, chest tubes were left in place for an average of 9.2 ± 4.8 days, which is about 3–4 days longer than times reported elsewhere for thoracoscopic talc pleurodesis [5, 26, 27]. This was attributable in part to surgeons insisting that chest tubes be removed as late as possible postoperatively. The National Health Service of Korea also enables low-cost hospitalization, encouraging longer stays. Further study is needed to directly compare thoracoscopic SIH with pleurodesis.

There were six patients of NR status and seven patients with recurrences. Pleural drainage was not repeated in one NR patient, who was not viewed as a recurrence. This patient had non-small cell lung cancer, with a left-sided MPE and malignant pericardial effusion. Chemotherapy was given for the primary cancer, once symptoms abated after thoracoscopic pericardial window and left-sided SIH. Unfortunately, both pericardial effusion and MPE recurred after 1 month. The patient was closely monitored without further drainage, given that the volume of fluid was lower than its initial level and did not increase further. Drainage of the pericardial effusion continued, but this patient died 44 days after surgery, suffering from acute renal failure and generalized edema.

Among the seven patients with recurrences, five had NR status, with one each achieving CR and PR. The patient of CR status underwent repeat bilateral drainage of pleural effusions, which recurred 6 months after SIH treatment for peritoneal cancer. At that time, the primary cancer had also recurred. Death occurred 2 months after re-drainage due to disease progression, although chemotherapy of the primary tumor was ongoing. The patient of PR status had common bile duct cancer and underwent repeat bilateral drainage of pleural effusions, which recurred 4 months after temporary improvement through SIH. The primary tumor was progressive, culminating in death 1 month after recurrence. All 17 patient mortalities during follow-up resulted from disease progression. Among the deaths were 13 responders to SIH, only two of which (15.4 %) experienced recurrences of pleural effusion. Recurrent effusions were actually few in number, underscoring that effects of SIH are not undermined by disease progression.

Five patients died early (within 3 months) due to disease progression—three of NR and two of PR status. None of the deaths were treatment-related. Risk factor analysis confirmed that NR status was a significant marker of rapid progression and early death. In other words, the efficacy of SIH is diminished by rapidly advancing disease, with life expectancy of < 3 months.

Several limitations of this study are acknowledged, the first being a retrospective analysis of operative results in a relatively small patient sampling at single center. Future studies with more cases may provide more accurate results, and a multicenter randomized trial may be required to validate our results. In addition, histologic studies after SIH were not done, and the basis (biologic and physiologic) for pleural hyperthermic effects remains unexplained. Although some have argued that tumor cells undergo apoptosis due to hyperthermia (shown in animal experimentation), this claim could not be corroborated here. In some patients for whom pleural biopsy was done intraoperatively (once or twice), no morphologic differences were detected in comparing histologic features of pleura before and after surgery. Undoubtedly, time is needed for apoptotic manifestations to materialize, so this observation is not unexpected. Still, it was not appropriate to re-biopsy patients under palliative treatment, merely to confirm tissue changes. Finally, evaluating the superiority of this therapy relative to other treatment methods proved difficult. Each study engaged a slightly different method, with no standard measures of treatment effect. A multicenter prospective study of MPE management might remedy this shortcoming.

## Conclusions

Although there are many ways to manage MPE, practical considerations and substantial side effects may restrict their usage. In patients for whom thoracoscopic exploration is indicated, SIH is one means of effectively managing MPE, provided the primary malignancy is not rapidly progressing. SIH is less rigorous than pleurodesis, with fewer side effects.

## Abbreviations

MPE: Malignant pleural effusion
SIH: Simple intrapleural hyperthermia
CR: Complete response
PR: Partial response
NR: Non-response
CT: Computed tomography
BP: Blood pressure
HR: Heart rate
TPC: Indwelling tunneled pleural catheter
